# Both kinds of RNase P in all domains of life: surprises galore

**DOI:** 10.1261/rna.068379.118

**Published:** 2019-03

**Authors:** Charles J. Daniels, Lien B. Lai, Tien-Hao Chen, Venkat Gopalan

**Affiliations:** 1 Department of Microbiology, The Ohio State University, Columbus, Ohio 43210, USA; 2 Center for RNA Biology, The Ohio State University, Columbus, Ohio 43210, USA; 3 Department of Chemistry and Biochemistry, The Ohio State University, Columbus, Ohio 43210, USA

**Keywords:** convergent evolution, RNase P, tRNA processing

## Abstract

RNase P, an essential housekeeping endonuclease needed for 5′-processing of tRNAs, exists in two distinct forms: one with an RNA- and the other with a protein-based active site. The notion that the protein form of RNase P exists only in eukaryotes has been upended by the recent discovery of a protein-only variant in Bacteria and Archaea. The use of these two divergent scaffolds, shaped by convergent evolution, in all three domains of life inspires questions relating to the ancestral form of RNase P, as well as their origins and function(s) in vivo. Results from our analysis of publicly available bacterial and archaeal genomes suggest that the widespread RNA-based ribonucleoprotein variant is likely the ancient form. We also discuss the possible genetic origins and function of RNase P, including how the simultaneous presence of its variants may contribute to the fitness of their host organisms.

## INTRODUCTION

Nonhomologous isofunctional enzymes are evolutionarily unrelated macromolecules catalyzing the same reaction, and encompass up to one-tenth of all biochemical transformations (Omelchenko et al. 2010). An example of such convergent evolution is presented by RNase P, a housekeeping endonuclease that functions primarily in 5′-maturation of tRNAs in all domains of life. RNase P is also the only nonhomologous isofunctional enzyme that has either an RNA- or a protein-based active site.

RNase P was first discovered more than four decades ago (Robertson et al. 1972) in a form that was subsequently found to be RNA-catalyzed (Guerrier-Takada et al. 1983). Present in all three domains of life, this ribonucleoprotein (RNP) form of RNase P exhibits considerable differences in size (∼135 to 400 kDa) due to a variable subunit composition: one catalytic RNA that is associated with one (Bacteria), up to five (Archaea), or as high as 10 (Eukarya) protein subunits (Altman 2007; Ellis and Brown 2009; Esakova and Krasilnikov 2010; Jarrous and Gopalan 2010; Lai et al. 2010). The existence of an RNA-free, proteinaceous RNase P (PRORP) was proven conclusively in 2008, after two decades of research (Holzmann et al. 2008; Pinker et al. 2012). PRORP consists of a ∼60-kDa protein functioning either alone or in association with two other protein partners (Holzmann et al. 2008; Gobert et al. 2010). Phylogenetic analysis of PRORP proteins indicated a presence in four of the five eukaryal supergroups, with Amoebozoa being the exception (Lechner et al. 2015). The domains Bacteria and Archaea were thought to be bereft of a proteinaceous RNase P form until recently when a polypeptide that could remove the 5′ leader from tRNAs in vitro was identified in the bacterium *Aquifex aeolicus* (Nickel et al. 2017). Sequences encoding this protein were found in a variety of bacterial and archaeal genomes, and the protein was named HARP (Homologs of Aquifex RNase P) (Nickel et al. 2017).

HARP is a 23-kDa protein identified by mass spectrometry as the most abundant protein in fractions exhibiting the highest activity during purification of *A. aeolicus* RNase P (Nickel et al. 2017). A recombinant form of this protein, obtained after overexpression in *Escherichia coli*, has RNase P activity in vitro under single- and multiple-turnover conditions (Nickel et al. 2017). Notably, HARP consists of only a metallonuclease domain, which is homologous to that of PRORP (Nickel et al. 2017). However, PRORP has in addition an N-terminal PPR domain that binds RNA substrates (Howard et al. 2013; Chen et al. 2016; Karasik et al. 2016; Pinker et al. 2017), raising the question of how HARP accomplishes substrate recognition. Moreover, both PRORP and HARP are members of the PIN domain-like superfamily. The PIN superfamily includes 100,000 proteins and constitutes a large, diverse family of exo- and endo-nucleases that are involved in RNA biogenesis as well as transcriptional or translational regulation (Matelska et al. 2017). This superfamily is divided into five major structural groups, each with multiple clusters. PRORP has its own cluster in the three-cluster PRORP group, whereas HARP belongs to the PIN_5 cluster of the VapC group that is comprised of 49 clusters. It remains to be determined how HARP and PRORP are evolutionarily related.

The unexpected finding that RNase P with either an RNA- or a protein-based active site exists in all three domains of life has spawned wide-ranging questions: Which form of RNase P supported the ancestral function in tRNA biogenesis? What are the possible genetic origins for these distinct RNase P scaffolds? What are the functional roles of the two forms in vivo, and are they redundant? These questions offer exciting research prospects.

## ANCESTRAL RNase P ACTIVITY LIKELY ACCOMPLISHED BY THE RNP FORM?

While the necessity for RNase P is due to the conservation of tRNA biogenesis pathways in all life (for more details, see McClain et al. 2010), the many guises of RNase P suggest that this need could be fulfilled through different but thematically overlapping catalytic strategies for hydrolysis of phosphodiester bonds. This idea is exemplified in the bacterial family Aquificaceae where Nickel et al. (2017) found six genomes, including *A. aeolicus*, to encode HARP but not the RNP form. They also found HARP encoded in a few other bacterial genomes and many more archaeal genomes, most of which also encode the RNP form. To better understand the full taxonomic distribution of HARP, we examined currently available bacterial and archaeal genomes using a combination of BLASTP and domain searches based on Pfam and COG assignments. We used previously reported HARP and the subunits of the RNP form in our searches (Rosenblad et al. 2006; Nickel et al. 2017). The presence of the bacterial RNase P protein RnpA and one or more of the archaeal RNase P proteins POP5, RPP21, RPP29, and RPP30 were used as proxies for the RNP form; our searches did not include the RNase P RNA. We recognize that many (95%) of the genome sequences are reported as “draft” status and that an apparent absence of a protein does not provide definitive evidence of its absence in a particular organism. Despite this limitation, in addition to confirming the earlier report of HARP's presence in Bacteria and Archaea (Nickel et al. 2017), some key inferences emerged from our comprehensive analysis.

**FIGURE 1.**
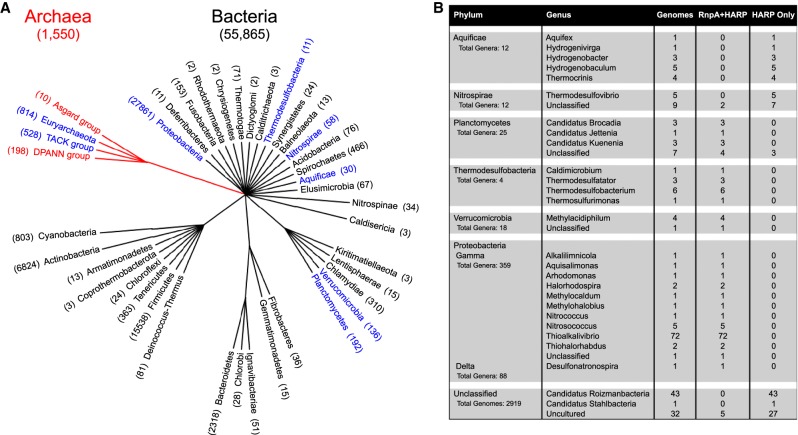
Occurrence of HARP in bacterial and archaeal genomes. HARP homologs were identified using BLASTP and domain searches at the National Center for Biotechnology Information (NCBI) and Integrated Microbial Genomes and Microbiomes (IMG/M). Established bacterial and archaeal HARP sequences (Rosenblad et al. 2006; Nickel et al. 2017) were used as queries in BLASTP searches. BLASTP matches (E ≤ 10^−20^ and bit scores >100) and members of the PIN_5/pfam08745, TIGR03875, or COG1458 families were selected as potential HARP homologs. Candidates identified using domain matches were verified by an independent BLASTP search. Genomes found to encode HARP were also examined for the occurrence of the RNase P RNP variant using as queries the bacterial RNase P protein RnpA and four archaeal RNase P proteins (POP5, RPP21, RPP29, and RPP30). (*A*) Taxonomic overview of HARP in bacterial and archaeal genomes. Bacteria are presented at the phylum level and Archaea as superphylum groupings (as described in Spang et al. 2017). Groups highlighted in blue have genomes encoding HARP. The number of genomes examined in each group is indicated in parentheses. (*B*) Occurrence of HARP in bacterial genera. This panel is an expanded view at the genus level of the number of genomes encoding HARP alone or in co-occurrence with RnpA; RnpA was detected in nearly all bacterial genomes. An additional 2919 unclassified bacterial genomes were examined for HARP and RNase P RNP proteins. These genomes are not assigned to any specific bacterial phyla and thus are not included in our taxonomic overview in *A*; however, some members encode potential HARP homologs, and are therefore listed in this panel. The number of genera in each phylum is provided to illustrate the limited presence of HARP within that phylum. The four archaeal RNase P proteins are absent from all bacterial genomes examined (data not shown). A full list of the genomes examined in this analysis is available at https://figshare.com/s/8089e9b333da30b69368.

First, HARP is encoded in only a few bacterial phyla in addition to Aquificae (Fig. 1A), and in most cases in only a small subset of the genera within each of these phyla (Fig. 1B). Of those bacterial genera with HARP, most have both HARP and RnpA. For example, only 10 of 359 genera in Gammaproteobacteria encode HARP, and all of them also encode the RNP form. An exception is found in Aquificae and Nitrospirae where a few of their genera encode only HARP. In addition, we detected the presence of HARP within unclassified groups of bacteria (Fig. 1B), some of which possess only HARP indicating that the HARP-only characteristic may be present in a few additional groups of organisms. The HARP-only scenario found in these bacteria provides a compelling argument for its function in tRNA maturation in these organisms.

**FIGURE 2.**
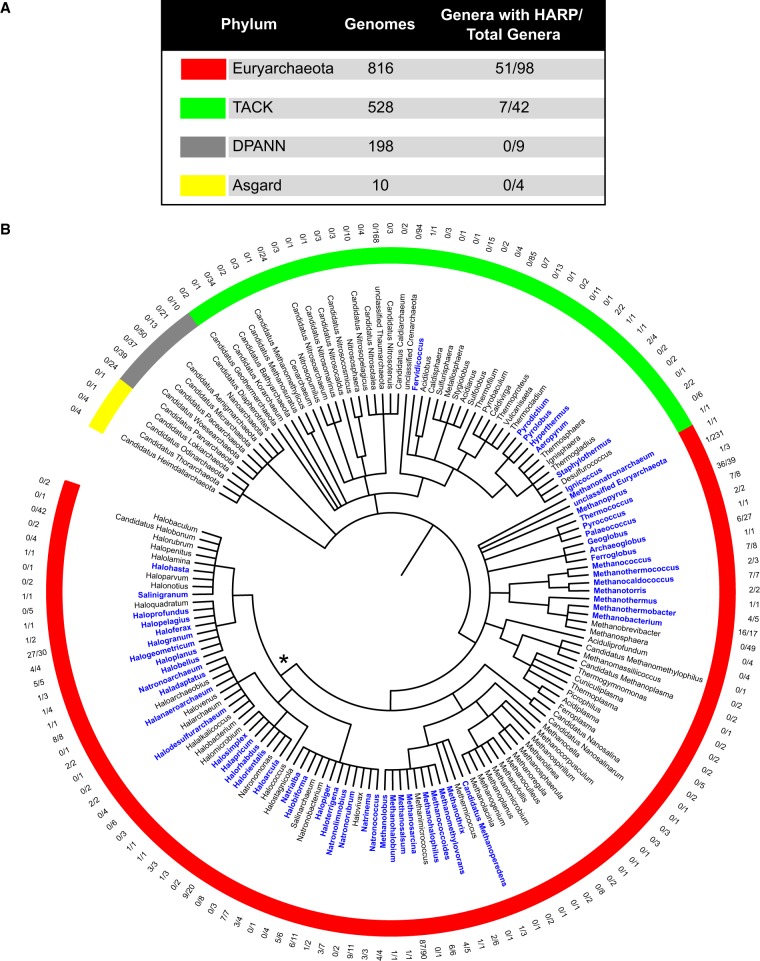
Presence of HARP in archaeal genomes. Genomes were queried for HARP as described in Figure 1 using BLASTP searches with known HARP sequences (Nickel et al. 2017), which have been identified as members of the PIN_5/pfam08745, TIGR03875, or COG1458 families. (*A*) Summary of the number of genomes and the fraction of genera whose genomes encode HARP in each archaeal superphylum. Most genomes are classified to the genus level, but many of the DPANN and Asgard organisms are described as Candidatus and were conditionally counted as genera in our analysis. Genomes were also queried for the bacterial RnpA and archaeal RNase P RNP proteins. The bacterial RnpA was not found in any archaeal genome examined (data not shown). All archaeal genomes encode one or more of the RNP proteins, indicating evidence for the RNP form (Samanta et al. 2016). (*B*) Analysis of archaeal genomes encoding HARP protein. Organisms are grouped taxonomically by their superphylum affiliation (indicated by the inner ring and mirroring the color scheme in *A*) and presented at the genus level. Genera encoding HARP are highlighted in blue with the fraction of HARP-containing genomes within each genus listed in the outer ring. The asterisk (*) denotes the class *Halobacteria* (see text for details).

Second, except for a few bacteria that encode only HARP, the RNP form is nearly universal in Bacteria and Archaea (Figs. 1 and 2). Indeed, a previous analysis of archaeal genomes indicated that all archaeal RNase P proteins are present in the majority of phyla, and support the hypothesis that the RNP form arose early in archaeal evolutionary history (Samanta et al. 2016). In contrast, among the archaeal genomes examined, we observed that HARP is present in a wide range of Euryarchaeota genera, but absent in many of the TACK and all of the DPANN and Asgard groups (Fig. 2A). In support of the universal occurrence of the RNP form in the Archaea (Samanta et al. 2016), we did not find any instance where HARP is present without the co-occurrence of one or more RNase P RNP proteins. The near-universal presence of the RNP variant in Bacteria and Archaea argues strongly for the RNP enzyme as the ancestral form.

Third, a closer look at HARP in Archaea revealed an interesting picture. HARP is widespread in Euryarchaeota and largely absent in the other groups (Fig. 2A). Within Euryarchaeota, there is a patchy distribution at the class level, which extends to the genus level in many cases (Fig. 2B). As an example, HARP is present in 26 of 46 genera within the class *Halobacteria* (denoted by the asterisk in Fig. 2B). While most of these genera contain species that are similar with respect to presence or absence of HARP, our data indicate that there are variations in many genera. For instance, in the genus *Haloarcula*, 9 of 20 genomes possess a HARP gene, but the two completed genomes of *Haloarcula hispanica* and *Haloarcula marismortui* do not have HARP. The patchy distribution of HARP in Euryarchaeota, together with its notable absence in the other archaeal groups (Fig. 2), is consistent with a horizontal gene transfer event, perhaps at the root of the Euryarchaeota branch.

## ROLE FOR HORIZONTAL GENE TRANSFER IN THE DISTRIBUTION OF HARP?

In all domains of life, catalysis by the RNP form of RNase P is dependent on the RNase P RNA (Guerrier-Takada et al. 1983; Pannucci et al. 1999; Kikovska et al. 2007). Given the high conservation of its active site embedded in a shared structural core, the origin of the RNase P RNA likely dates back to the last universal common ancestor as may be expected of a remnant from the RNA world (Gopalan 2007; Ellis and Brown 2009; Lai et al. 2010). HARP (and possibly PRORP), in contrast, may have a more checkered history.

Nickel et al. (2017) postulated that *A. aeolicus* acquired HARP by horizontal gene transfer based on their finding of HARP's proximity in the genome to a resident archaeal RNase P protein homolog; such exchange of genetic material between bacterial hyperthermophiles like *A. aeolicus* and archaea sharing the same environmental niche has been well recognized (Aravind et al. 1998). However, we were unable to identify any archaeal RNase P protein in *A. aeolicus* or other bacterial genomes examined; likewise, we did not detect the bacterial RNase P protein RnpA in any archaea. Thus, the origin of HARP itself is an intriguing question.

An attractive hypothesis is that HARP originated from a toxin–antitoxin system, whose mobility by horizontal gene transfer is well documented (Yamaguchi et al. 2011; Page and Peti 2016). As HARP is a PIN protein akin to PRORP, it is interesting to note that one-fifth of all PIN domain-like superfamily proteins in Bacteria and Archaea are involved in toxin–antitoxin systems (Matelska et al. 2017). A toxin acts by inhibiting an essential cellular process, but is sequestered in a complex with an antitoxin until specific cues (e.g., nutrient deprivation) lead to degradation of the antitoxin. Diverse and widespread toxin–antitoxin loci are present in chromosomes, plasmids, and phages, and contribute to growth arrest and persistence of microbes (Yamaguchi et al. 2011; Page and Peti 2016). There is a nexus to the RNA world given that three of the six toxin–antitoxin systems entail the use of an RNase as a toxin (Page and Peti 2016).

While there are several ideas as to how toxin–antitoxin systems help bacteria and archaea persist during crises and therefore engender this toxin–antitoxin dependency, one could envision how genetic drift repurposed some of these toxin RNases. For instance, if HARP progenitors were RNases with sequence-/structure-recognition determinants for cleavage of select RNAs, it is conceivable that site-specific 5′-processing of pre-tRNAs (and other cellular RNAs) could have been achieved with modest reprogramming especially if HARP started out with a substrate suite resembling some aspects of pre-tRNAs. Determining the extant cellular substrates of HARP may uncover such an ancestral role and reveal the full scope of HARP functions. While HARP appears to singly fulfill a role in tRNA processing in some bacteria (e.g., *Aquifex*), if it had an early origin then it is unclear why the near-universal RNP form of the enzyme has supplanted it. A more parsimonious explanation is that the RNP form, with its origin in the RNA world, is likely the ancient version. Regardless of their antiquity, why have two forms of RNase P persisted and how do they function when both are present?

## NECESSITY FOR TWO FORMS OF RNase P: DISTRIBUTED ROBUSTNESS?

While Nickel et al. (2017) have shown that HARP has RNase P activity in vitro, the expression and biological function of HARP remain to be demonstrated in vivo, especially in organisms where it is present with the RNP form. If indeed HARP contributes to RNase P function in these species, redundancy offers a safety net for a structure or activity that is essential for life. The lack of lethality upon knocking out the sole nuclear PRORP variant in the moss *Physcomitrella patens* led to the idea that a yet unidentified RNP form might function in the nucleus and provide a back-up (Sugita et al. 2014). If proven, this would represent a situation where both forms of RNase P co-exist and provide safeguards within a single compartment. Another advantage may be relevant when one considers the RNP form in the nucleus and PRORP in the mitochondria of eukaryotic cells, with the latter presumably affording gains with respect to transport and assembly (Lai et al. 2010). Other perspectives including nonoverlapping functions (e.g., distinct suites of substrates) also merit consideration given that both forms are present in many archaeal and a few bacterial species (Figs. 1 and 2).

In the euryarchaeon *Methanothermobacter thermautotrophicus*, both forms are encoded in the genome and the respective recombinant forms are functional in vitro (Li et al. 2009; Chen et al. 2010; Nickel et al. 2017). If both are expressed, the RNP form, which displays up to 100-fold higher activity in vitro compared to HARP (Li et al. 2009; Chen et al. 2010; Nickel et al. 2017), is likely better suited to process pre-tRNAs during rapid growth on nutrient-rich conditions. On the other hand, expression of HARP under suboptimal conditions might help decrease the synthesis cost—instead of a six-subunit ∼200-kDa RNP, a 23-kDa HARP (even as a 69-kDa trimer [Nickel et al. 2017]) might suffice for biogenesis of tRNA and other noncoding RNAs during these stress conditions.

If indeed HARP originated as a toxin gene, repurposing this gene may have promoted its retention. For example, if environmental or stress conditions led to inhibition or decrease of the RNP-based RNase P activity, then HARP may have permitted a low level of tRNA biogenesis sufficient for a “persister” state until relief of the initial stress. Genetic studies could test the conjecture that having both forms confers distributed robustness (Wagner 2005).

What about scenarios where HARP is present exclusively as in the case of *A. aeolicus*? HARP must be adequate to support tRNA biogenesis even under conditions requiring high tRNA levels, unless there is another (yet unidentified) form of RNase P. But if HARP alone suffices in some bacteria, why has the RNP form not been completely replaced in Archaea and Eukarya? Because subunits in archaeal and eukaryotic RNase P RNP are multifunctional (Gopalan et al. 2018), replacing the RNP with HARP/PRORP would not afford any economy with respect to synthesis of the biocatalytic repertoire. In fact, replacement of the yeast RNase P RNP by HARP or PRORP resulted in complementation but weaker growth after a (several-day) lag (Taschner et al. 2012; Weber et al. 2014; Nickel et al. 2017). While any positive result in these challenging genetic studies is remarkable, the slower growth might indicate, among other reasons, a catalytic insufficiency of the protein-based form compared to its RNP counterpart.

## SUMMARY

The discovery of HARP has inspired questions pertaining to the form and function of the two divergent RNase P scaffolds sculpted by convergent evolution, as well as to the indispensability of the RNP variant in the protein-dominated cellular milieu. Despite the growing recognition that parsing essential or nonessential attributes of any gene requires testing under various biotic and nonbiotic stress conditions (Hillenmeyer et al. 2008), modern genetic retrofitting methods lend promise to assessing if mere chance or nonoverlapping functional repertoires (with possible fitness gains) dictated the provenance and distribution of the nonhomologous isofunctional enzyme forms of RNase P.
